# Overlapping Phenotypes Associated With *CYP24A1*, *SLC34A1*, and *SLC34A3* Mutations: A Cohort Study of Patients With Hypersensitivity to Vitamin D

**DOI:** 10.3389/fendo.2021.736240

**Published:** 2021-10-13

**Authors:** Arnaud Molin, Sandrine Lemoine, Martin Kaufmann, Pierre Breton, Marie Nowoczyn, Céline Ballandonne, Nadia Coudray, Hervé Mittre, Nicolas Richard, Amélie Ryckwaert, Alinoe Lavillaureix, Glenville Jones, Justine Bacchetta, Marie-Laure Kottler

**Affiliations:** ^1^ Caen University Hospital, Department of Genetics, Molecular Genetics Laboratory and Reference Center for Rare Diseases of Calcium and Phosphorus Metabolism (OSCAR), Caen, France; ^2^ Caen Normandy University, Medical School, Caen, France; ^3^ BioTARGEN, Caen Normandy University, Caen, France; ^4^ OeReCa, Caen Normandy University, Caen, France; ^5^ Department of Nephrology and Renal Functional Explorations, Edouard Herriot Hospital, Lyon, France; ^6^ University of Lyon, University of Lyon 1, Villeurbanne, France; ^7^ Queen’s University, Department of Biomedical and Molecular Sciences, Kingston, ON, Canada; ^8^ Caen University Hospital, Department of Biochemistry, Caen, France; ^9^ Department of Pediatrics, Rennes University Hospital, Rennes, France; ^10^ Department of Genetics, Rennes University Hospital, Rennes, France; ^11^ Reference Center for Rare Kidney Diseases (ORKID), Department of Pediatric Nephrology, Rhumatology and Dermatology, Woman Mother Children Hospital, Bron, France; ^12^ Reference Center for Rare Diseases of Calcium and Phosphorus Metabolism (OSCAR), Department of Pediatric Nephrology, Rhumatology and Dermatology, Woman Mother Children Hospital, Bron, France; ^13^ INSERM 1033, Bone Diseases Prevention, Lyon, France

**Keywords:** hypersensitivity to vitamin D, calcitriol induced hypercalcemia, phosphate wasting diseases, vitamin D, *CYP24A1*- hydroxylase, *SLC34A1* gene, *SLC34A3* gene

## Abstract

Mutations in *CYP24A1* (vitamin D 24-hydroxylase) and *SLC34A1* (renal phosphate transporter NPT2a) cause autosomal recessive Infantile Hypercalcemia type 1 and 2, illustrating links between vitamin D and phosphate metabolism. Patients may present with hypercalciuria and alternate between chronic phases with normal serum calcium but inappropriately high 1,25-(OH)_2_D and appropriately low PTH, and acute phases with hypercalcemia with suppressed PTH. Mutations in *SLC34A3* and *SLC9A3R1* have been associated with phosphate wasting without hypercalcemia. The aims of this study were to evaluate the frequency of mutations in these genes in patients with a medical history suggestive of *CYP24A1* mutation to search for a specific pattern. Using next generation sequencing, we screened for mutations in 185 patients with PTH levels < 20 pg/mL, hypercalcemia and/or hypercalciuria, and relatives. Twenty-eight (15%) patients harbored biallelic mutations in *CYP24A1* (25) and *SLC34A3* (3), mostly associated with renal disease (lithiasis, nephrocalcinosis) (86%). Hypophosphatemia was found in 7 patients with biallelic mutations in *CYP24A1* and a normal phosphatemia was reported in 2 patients with biallelic mutations in *SLC34A3*. Rare variations in *SLC34A1* and *SLC34A3* were mostly of uncertain significance. Fifteen patients (8%) carried only one heterozygous mutation. Heterozygous relatives carrying *SLC34A1* or *SLC34A3* variation may present with biochemical changes in mineral metabolism. Two patients’ genotype may suggest digenism (heterozygous variations in different genes). No variation was found in *SLC9A3R1.* As no specific pattern can be found, patients with medical history suggestive of *CYP24A1* mutation should benefit from *SLC34A1* and *SLC34A3* analysis.

## Introduction

Hypersensitivity to vitamin D (HVD) may be defined as an inability to regulate vitamin D metabolism which leads to calcium homeostasis deregulation, especially in case of vitamin D supplementation (1). This phenotype was classically associated with Idiopathic Infantile Hypercalcemia (IIH), a condition firstly described in the early fifties (2). Children present with non-specific signs of hypercalcemia (failure to thrive, vomiting, polyuria and polydipsia) with appropriately low parathormone (PTH) and inappropriately high 1,25-(OH)_2_D, suggesting deregulation of vitamin D metabolism.

In 2011, mutations in *CYP24A1* encoding the vitamin D 24-hydroxylase were identified as the cause of Infantile Hypercalcemia type 1 (IH1) (MIM 143880) with HVD phenotype (3, 4). This enzyme is responsible for inactivating 1,25-(OH)_2_D and 25-OH-D (5–7). A high 25-OH-D_3_:24,25-(OH)_2_D_3_ ratio (substrate over product ratio, R ratio), which directly reflects the enzymatic defect, has been specifically associated with this condition and proposed as an effective screening tool (8–10). The clinical spectrum has been broadened to older children, teenagers and adults with nephrocalcinosis, renal stones and chronic fluctuating high serum and urine calcium with decreased serum PTH (9, 11–15). Patients present with a lifelong susceptibility to acute hypercalcemia and hypercalciuria, especially in case of vitamin D supplementation (16) and sunshine exposure (17).

In 2016, mutations in *SLC34A1*, encoding the renal phosphate transporter NPT2a, were reported as the cause of Infantile Hypercalcemia type 2 (IH2) (MIM 616963) with renal phosphate wasting and hypophosphatemia (18). A few cases have been published thereafter with clinical phenotype similar to type 1 (19–21). These observations suggest that phosphate wasting disorders may lead to HVD.

Other genes have been associated with renal phosphate wasting. Mutations in *SLC34A3* encoding the renal phosphate transporter NPT2c have been associated with autosomal recessive hypophosphatemic rickets (ARHR) with hypercalciuria (MIM 241530) (22, 23). Its phenotype also includes biochemical features of HVD (low PTH, high serum 1,25-(OH)_2_D, high urine calcium with renal stones and nephrocalcinosis) and normal to low FGF23 serum concentrations. Furthermore, two patients with idiopathic hypercalciuria without bone signs and biallelic variations in *SLC34A3* were described suggesting a potential role of *SLC34A3* in HVD phenotype (24). Lastly, *SLC9A3R1*, encoding NHERF1 (sodium-proton exchanger regulatory factor 1) protein, was previously associated with autosomal dominant phosphate wasting (25, 26). This protein is implicated in transport regulation of NPT2a to the apical membrane of the proximal tubular cells and control of its retrieval by PTH (27).

We have previously published a cohort of 72 patients (1-72, **Supplementary Table S1**) with HVD tested for *CYP24A1* mutations (9). We highlighted a wider phenotype depending on vitamin D intakes, from a balanced state with normal or high normal serum calcium and hypercalciuria to an unbalanced state with hypercalcemia and hypercalciuria, but consistent low PTH. In the present study, we aimed to evaluate the respective contribution of above mentioned genes involved in phosphate wasting in this cohort enriched with 113 new patients. Our objective was to search for a diagnostic algorithm to guide candidate gene testing in patients with features of HVD.

## Patients and Methods

### Patients

Over a 5 years period, we enrolled for molecular analysis 185 patients (index cases) presenting with a serum PTH concentration <20 pg/mL (2^nd^ generation PTH assay were considered, normal values: 15-65 pg/mL) and a medical history of acute or chronic hypercalcemia (>2.6 mmol/L) and/or chronic hypercalciuria. Patients with medical history of calcium homeostasis deregulation with PTH > 20 pg/mL were not considered.

Recruitment was national through the network of the National Center for rare diseases of calcium and phosphate metabolism.

After genetic counseling, relatives of patients carrying biallelic variations were studied for genetic screening. Clinical and biochemical data were available in 17 relatives.

Written informed consent was obtained from the patients and/or their parents for retrospective collection of clinical and laboratory data, and for DNA collection to conduct molecular studies.

### Biochemical and Clinical Parameters

Data on clinical symptoms were collected retrospectively using records from hospitals or primary care physicians.

Data on biochemical parameters (calcium and phosphate in serum and urine, renal function: creatinine and estimation of glomerular filtration rate with CKD-EPI for adults (28), PTH and vitamin D concentrations) were collected retrospectively using the same procedure, and thus correspond to a collection of different methods from many clinical chemistry departments. Approximate normal values for 1,25-(OH)_2_D were used as follows considering the use of different assays inherent in such retrospective study: 65-134 pmol/L for children and 60-108 pmol/L for adults (29). As most of routine assays for vitamin D cannot discriminate between vitamin D_3_ and D_2_, we used the term “vitamin D” to describe these measurements. As serum phosphate decreases from birth to adulthood, values were expressed as Z-scores to avoid the effects of the age-related variations of reference values (24, 30, 31).

Routine biochemical assays were mostly performed at the time of the diagnosis of acute hypercalcemia, or during follow up after normalization of the calcium level.

For urinary calcium: creatinine ratio, the different cutpoints of normal level were as follow: adults <0.33; children under 6 months <2.4; children 7-12 months <1.7; children 1-5 years <1.1 and children above 5 years <0.7.

Liquid Chromatography Tandem Mass Spectrometry (LC-MS/MS) analysis was performed as previously described using 100 µL of serum. Results were expressed as the molar ratio of 25-OH-D_3_: 24,25-(OH)_2_D_3_ (R ratio) (8). Values under 25 indicated no defect in 25-OH-D_3_-24-hydroxylase activity and were considered as normal.

Medullary nephrocalcinosis was assessed by renal ultrasonography (echogenic renal pyramids). Nephrolithiasis was defined as kidney stones on ultrasonography or a medical history of iterative renal colic.

### Molecular Analysis

Genomic DNA was extracted from whole blood samples with QuickGene DNA Whole Blood kit (Kurabo Industries LTD, Osaka, Japan) and QuickGene-610L extraction system (Fujifilm LTD, Singapore, Republic of Singapore).

Coding exons and flanking regions of *CYP24A1* (NM_000782.4), *SLC34A1* (NM_003052.4), *SLC34A3* (NM_001177317.1) and *SLC9A3R1* (NM_004252.4) genes were studied by Massively Parallel Sequencing (MPS) using a custom Ampliseq library on the Ion Torrent Personal Genome Machine (PGM) (ThermoFisher Scientific, Waltham, Massachusetts, USA). Reads were aligned against the reference sequences and variants were annotated using IonSuit (ThermoFisher Scientific, Waltham, Massachusetts, USA) and Nextgene (SoftGenetics, State College, Pennsylvania, USA). A read depth (RD)-based approach was used to detect copy number variation (CNV) from targeted MPS data using the CovCop tool (32).

Sequences with coverage <30X and variations identified with MPS were studied with Sanger sequencing using Big Dye^®^ Terminator v1.1 Cycle Sequencing kit (ThermoFisher Scientific, Waltham, Massachusetts, USA) on a ABI 3500 Sequencer (ThermoFisher Scientific, Waltham, Massachusetts, USA).

Variations of sequence were searched on Varsome database. Interpretation was based on standards and guidelines of the American College of Medical Genetics (33). Pathogenicity prediction programs PolyPhen-2, Align-GVGD, MutationTaster and SIFT were considered for *in silico* analyses. Allelic frequencies were evaluated through the GnomAD database. For research purposes, variations classified as of uncertain significance (III) were considered apart from mutations [i.e. likely pathogenic (IV) and pathogenic (V) variations] as some may represent pathogenic mutations and other benign variations.

### Statistical Analysis

Data from patients with class IV and V variations were used to compare biochemical profiles.

Statistical analyses were made using Prism 7 (GraphPad Software Inc.). For each biochemical parameter, differences between groups were calculated using the Mann and Whitney nonparametric U test. A two-tailed p value <0.05 was considered statistically significant. Kruskal-Wallis test was used for multiple group comparison (one-way nonparametric ANOVA).

Sex was not considered a factor in the statistical analysis of the data.

Chi-squared test was used to compare the allelic frequency of recurrent variations in our cohort and in the general population (estimated from data of GnomAD database).

## Results

### Patients

We enrolled 185 patients [88 males (47%) and 97 females (53%)], including 150 children (81%) (range: 1 day – 16 years; mean 2.3 years) and 35 adults (19%) (range: 18-81 years; mean 42 years). Familial occurrence was recorded in 34 patients (18%).

#### Molecular Analysis: Variations

The frequency and the criteria in favor of pathogenicity of each variation are presented in **Table 1**.

**Table 1:** Description of genetic variations which were identified in *CYP24A1*, *SLC34A1* and *SLC34A3*.

Gene	Variation type	rs	f gnomAD	hmz	f all max (pop)	Exon	c.	p.	Pathogenic impact	Benign impact	Variants class ACMG	Publication
*CYP24A1*	frameshift	774432244	0.0000626	0	0.0000626 (European non Finnish)	exon 1	c.62del	p.(Pro21Argfs*8)	PVS1, PM2, PM3, PP3		V	(9)
	frameshift	–	0.000004314	0	0.0001922 (other)	exon 1	c.233del	p.(Gly78Valfs*22)	PVS1,PM2,PM3, PP3		V	
	in frame	777676129	0.0005445	1	0.001065 (European non Finnish)	exon 2	c.427_429del	p.(Glu143del)	PS3, PS4, PM3, PM4, PP5		V	(9)
	missense	139763321	0.0001585	0	0.001827 (Ashkenazi Jewish)	exon 2	c.443T>C	p.(Leu148Pro)	PM3, PP1, PP3, PP5		IV	(9)
	nonsense	14718997603	0.000004063	0	0.00003249 (South Asian)	exon 3	c.464G>A	p.(Trp155*)	PVS1, PM2, PP3		V	(9)
	missense	–	–	–	–	exon 6	c.758T>A	p.(Met253Lys)	PS3, PM2, PM3, PP3		IV	(9)
	missense	–	–	–	–	exon 7	c.965A>C	p.(Glu322Ala)	PS3, PM2, PM5, PP3		IV	(9)
	missense	1224687481	0.00003236	0	0.0001152 (African)	exon 7	c.980C>T	p.(Ala327Val)	PS3, PM2,PM3, PP3		IV	–
	missense	552310427	0.00007715	0	0.001117 (Ashkenazi Jewish)	exon 7	c.989C>T	p.(Thr330Met)	PS3, PM2,PM3, PP3		IV	(9)
	frameshift	–	–	–	–	exon 8	c.1003dup	p.(Leu335Profs*11)	PVS1, PM2, PM3, PP3		V	(9)
	missense	–	–	–	–	exon 8	c.1138T>C	p.(Cys380Arg)	PS3, PM2,PM3, PP3		IV	(9)
	missense	114368325	0.0006825	1	0.001399 (European Finnish)	exon 9	c.1186C>T	p.(Arg396Trp)	PS3, PS4,PM3, PP3, PP5		V	(9)
	missense	143934667	0.00007317	0	0.0001523 (European non Finnish)	exon 9	c.1187G>A	p.(Arg396Gln)	PS4, PM2, PM3, PM5, PP3		V	(9)
	missense	–	–	–	–	exon 9	c.1193T>C	p.(Leu398Pro)	PS3,PM2,PM3		IV	
	frameshift	–	–	–	–	exon 9	c.1206del	p.(Val403Phefs*15)	PVS1, PM2, PM3, PP3	BP4	V	(9)
	missense	6068812	0.0007687	0	0.001371 (European non Finnish)	exon 9	c.1226T>C	p.(Leu409Ser)	PS3, PS4, PM3, PP3, PP5		V	(9)
	missense	374292194	0.00004469	0	0.0001825 (other)	exon 10	c.1315C>T	p.(p.Arg439Cys)	PS3, PM2, PP3		IV	(9)
	missense	748429181	0.000008123	0	0.00001791 (European non Finnish)	exon 10	c.1366G>C	p.(Gly456Arg)	PM2, PP3		III	(9)
	nonsense	988715134	0.000008123	0	0.00002978 (Latino)	exon 10	c.1396C>T	p.(Arg466*)	PVS1, PM2, PP3		V	(34)
	frameshift	–	–	–	–	exon 10	c.1406_1407del	p.(Glu469Alafs*22)	PVS1, PM2, PM3		V	(9)
	large deletion	–	–	–	–	exon 10-12	g.52763705_52774628del	–	–		V	(9, 35)
*SLC34A1*	in frame	876661296	0.01696	36	0.02726 (European Finnish)	exon 4	c.272_292del	p.(Val91_Ala97del)	PS3, PM3, PM4, PP5	BS2	III	(18)
	missense	–	–	–	–	exon 6	c.578T>A	p.(Ile193Asn)	PM2, PM3, PP3		III	
	splicing?	200095793	0.00006499	0	0.0002599 (South Asian)	intron 9	c.1006+1G>A	p.?	PVS1, PS3, PM2, PP3, PP5		V	(18)
	missense	369749329	0.00008971	0	0.00008971 (European non Finnish)	exon 12	c.1352C>T	p.(Thr451Ile)	PM2, PP3		III	
	splicing?	376448083	0.000354	0	0.0006564 (European non Finnish)	Intron 12	c.1416+3G>A	p.?		BP4	III	
	splicing?	202081023	0.0005237	0	0.0008868 (European non Finnish)	intron 12	c.1416+5G>A	p.?	PP5	BP6	III	(18)
	missense	756685605	4.07e-06	0	0.000008967 (European non Finnish)	exon 13	c.1466A>G	p.(Tyr489Cys)	PM2, PP3		III	
	missense	–	–	–	–	exon 13	c.1588G>A	p.(Val530Met)	PM2		III	
	missense	756519888	0.000008126	0	0.000008957 (European non Finnish)	exon 13	c.1645G>A	p.(Gly549Arg)	PM2		III	
*SLC34A3*	deletion	532224704	0.000965	0	0.004758 (African)	exon 4	c.195_215del	p.(Arg65_Gly71del)	PM4	BP6	III	
	splicing?	201293634	0.00003666	0	0.00007199 (European Finnish)	intron 4	c.304+2T>C	p.?	PVS1, PM2, PP3, PP5		V	(22)
	missense	200536604	0.00004483	0	0.00008941 (Latino)	exon 6	c.496G>A	p.(Gly166Ser)	PM2, PP3		III	
	frameshift	–	–	–	–	exon 7	c.578_579insT	p.(Val194Glyfs*28)	PVS1, PM2	BP4	IV	
	deletion	–	1.31e-05	0	0.0001961 (other)	intron 9	c.925+20_926-48del	p.?	PVS1, PS3, PM2, PM3, PP5		V	(23, 36)
	missense	1405547154	0.00001688	0	0.00004061 (South Asian)	exon 10	c.926G>A	p.(Cys309Tyr)	PM2		III	
	frameshift	1473689787	6.56e-05	0	0.0001355 (European Finnish)	exon 10	c.944del	p.(Gly315Alafs*28)	PVS1, PM2	BP4	III	
	frameshift	–	–	–	–	exon 10	c.1055_1058dup	p.?	PVS1, PM2, PM3		V	
	frameshift	–	–	–	–	exon 10	c.1058_1065dup	p.?	PVS1, PM2		IV	
	missense	748862410	0.00002149	0	0.00004769 (European non Finnish)	exon 11	c.1198G>C	p.(Val400Leu)	PM2, PM3		III	
* *	missense	771932709	0.00002827	0	0.00002827 (European non Finnish)	exon 11	c.1207A>G	p.(Met403Val)	PM2	BP4	III	
	missense	532292902	0.00006341	0	0.0001889 (other)	exon 11	c.1208T>G	p.(Met403Arg)	PM2, PM3, PP3		III	
	missense	775653752	0.000004093	0	0.000009047 (European non Finnish)	exon 12	c.1283C>T	p.(Ala428Val)	PM2, PP3		III	
	missense	772211127	0.0000165	0	0.00006619 (African)	exon 13	c.1369G>A	p.(Gly457Ser)	PM2, PM3, PP5		III	(24)
	missense	149389629	0.0001118	0	0.0002501 (European non Finnish)	exon 13	c.1462G>C	p.(Ala488Pro)	PM3, PP3		III	

ACMG classification: III: variation of uncertain significance; IV: likely pathogenic variation; V : pathogenic variation.

f gnomAD: allelic frequency reported in exome gnomAD database; f all max (pop): maximum allelic frequency reported in exome gnomAD database (population with maximum allelic frequency); hmz: number of subjects carrying the variation in a homozygous state reported in exome gnomAD database; UTR: untranslated region.

We identified 20 mutations in *CYP24A1*, 1 mutation in *SLC34A1* and 6 mutations in *SLC34A3*. Adding class III variations, the number of variations rose to 21 variations in *CYP24A1*, 9 variations in *SLC34A1* and 15 variations in *SLC34A3*.

We described 2 new missenses pathogenic variations c.980C>T/p.(Ala327Val) and c.1193T>C/p.(Leu398Pro) in patients with elevated R ratio.

The recurrent variation in *SLC34A1* c.272_292del/p.Val91_Ala97del (REC DEL in **Supplementary Table S1**) was identified in 10 patients (**Table 1**). In our cohort, its allelic frequency is 2.7%. A Chi-squared test showed that this frequency was not statistically different from the allelic frequency found in general population (1.696% in general population, 2.537% in European non-Finnish population according to GnomAD Exomes database).

#### Molecular Analysis: Genotype

Patients were classified into different groups according to their genotype (gene with variation, biallelic, i.e. homozygous or compound heterozygous, or monoallelic, i.e. heterozygous) (**Table 2**).

**Table 2:** Genotype in the cohort.

	IV/V			III/IV/V		
	children	adults	total	children	adults	total
No variation	126	16	142 (77)	110	12	122 (66)
Biallelic variation	12	16	28 (15)	18	18	36 (19)
*CYP24A1*	9	16	25 (14)	10	16	26 (14)
*SLC34A1*	0	0	0 (0)	3	1	4 (2) [2]
*SLC34A3*	3	0	3 (2)	5	1	6 (3)
Monoallelic variation	12	3	15 (8)	20	5	25 (14)
*CYP24A1*	7	2	9 (5)	6	2	8 (4)
*SLC34A1*	1	0	1 (1)	9	2	11 (6) [7]
*SLC34A3*	4	1	5 (3)	5	1	6 (3)
Digenic patients	0	0	0 (0)	2	0	2 (1) [1]
TOTAL	150	35	185	150	35	185

Depending on the inclusion of class III variations, we identified biallelic variations in 28-36 patients (15-19%) and monoallelic variations in 15-25 patients (8-14%) (heterozygous status) and no variation was found in 122-142 patients (66-77%) patients. Variations in *CYP24A1* (biallelic as well as monoallelic) are more frequently found (18-19%) followed by those in *SLC34A1* (1-8%) and *SLC34A3* (5-6%). No variation was found in *SLC9A3R1*.

#### Digenic Patients

Two patients presented with one heterozygous class III *SLC34A3* and a second variation in *SLC34A1* (recurrent deletion) or *CYP24A1* (the recurrent pathogenic variation p.Leu409Ser).

**Figure 1:**
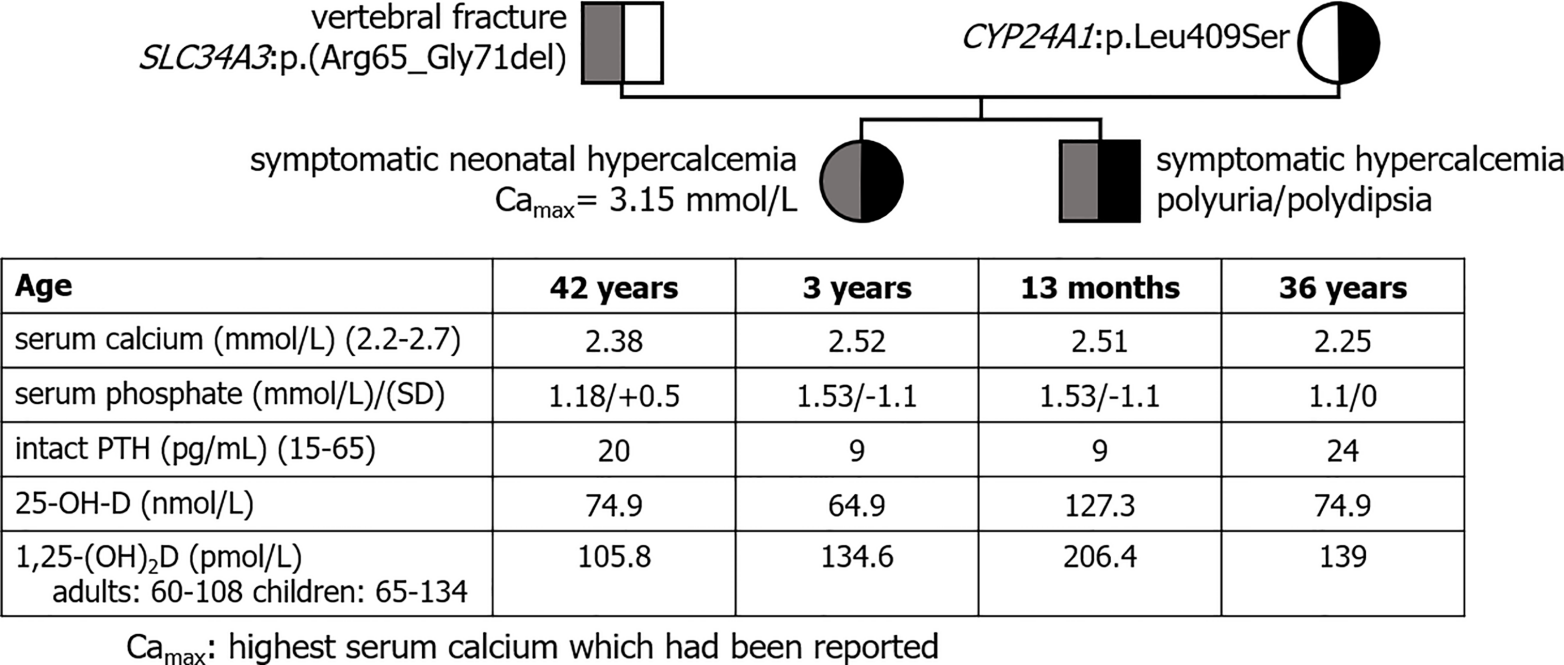
Pedigree tree of a family with suspected mechanism of digenism. Both children shared a biochemical profile evocative of HVD. Both parents shared a biochemical profile with normal serum calcium, high 1,25-(OH)_2_D, low PTH and a normal 25-OH-D evocative of mild HVD.

Especially, we reported 2 children of a non-consanguineous healthy couple who had experienced symptomatic neonatal hypercalcemia with similar biochemical profile (**Figure 1**). Both carried a *SLC34A3* variation of uncertain significance inherited from their father and a *CYP24A1* recurrent mutation inherited from their mother. The father presented with vertebral non-traumatic fracture; the mother was healthy.

#### Phenotype of Patients With Biallelic Variation

Biallelic variations were found with a higher frequency in adult patients (45-51%; n= 16-18/35) than in children (8-12%; n= 12-18/150), most under 2 years (**Table 2**).

In patients with nephrocalcinosis (n=55; 44 children and 11 adults), we identified 18 (33%) patients with biallelic class IV or V variations, mostly in *CYP24A1* (n=25/28) and also in *SLC34A3* (n=3/28); 4 adults with biallelic *CYP24A1* mutations presented chronic kidney disease (CKD-EPI<60mL/min). In patients with renal lithiasis (n=28; 18 children and 10 adults), we identified 6 (21%) patients with biallelic mutations in *CYP24A1*, mostly adults (n=5/6). Of note, *CYP24A1* biallelic mutations were also found in 4 adult patients with medical history of hypercalcemia without nephrocalcinosis nor renal stones.

One patient was referred at the age of 5 for hypercalciuria and nephrocalcinosis, normal serum calcium and low PTH. At the age of 10, bowing of the lower limbs was evocative of rickets. He harbored a homozygous *SLC34A3* mutation inherited from his consanguineous parents. His mother and his two brothers with the same mutation in a heterozygous state also presented nephrocalcinosis and no signs of bone disease.

Including class III variations, we identified 3 more patients with biallelic variations in *SLC34A3* and nephrolithiasis (n=1) or nephrocalcinosis (n=2) and 4 patients with biallelic *SLC34A1* variations, 2 of them (patients 103 and 170) having nephrocalcinosis detected as hyperechogenic kidney *in utero* (21).

**Figure 2:**
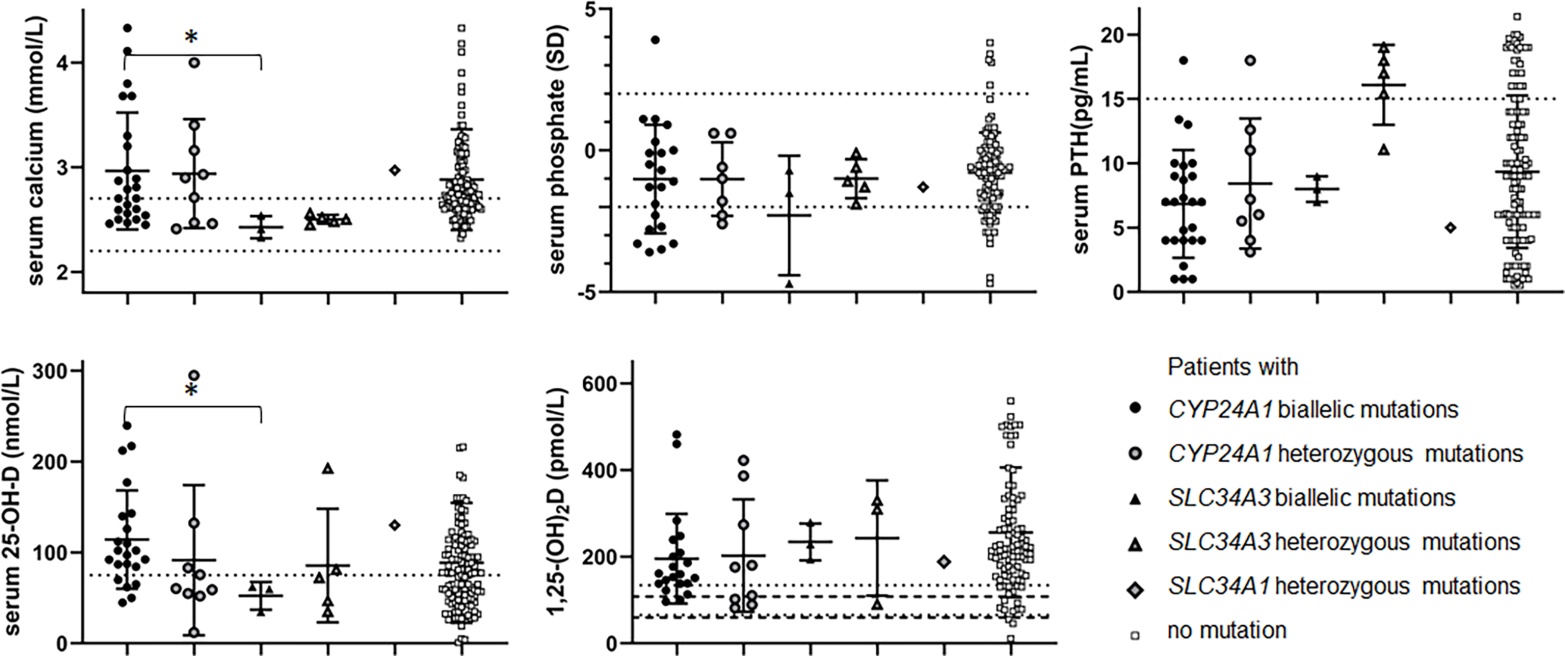
Biochemical data according to genotype: serum calcium (mmol/L), serum phosphate (SD), PTH (pg/mL) 25-OH-D (nmol/L) and 1,25-(OH)_2_D (pmol/L). Black dot: patients with biallelic *CYP24A1* mutations. Grey dot: patients with heterozygous *CYP24A1* mutations. Black triangle: patients with biallelic *SLC34A3* mutations. Grey triangle: patients with heterozygous *SLC34A3* mutations. Grey diamond: patients with heterozygous *SLC34A1* mutations. White square: patients without variation. Serum calcium dot lines: normal values 2.2-2.7 mmol/L. Serum phosphate dot lines: -2-+2SD. Serum PTH dot line: 15 pg/mL. 25-OH-D dot line: 75 nmol/L. 1,25-(OH)_2_D dot and hyphen lines: approximative normal values for children and adults. *p ≤ 0.05.

Hypophosphatemia (serum phosphate Z-score < -2) was reported in 7 patients (27-28%) with *CYP24A1* biallelic mutations and 1 patient with *SLC34A3* biallelic mutations (**Figure 2**, **Table 3** and **Supplementary Table S1**).

**Table 3:** Biochemical data according to genotype.

n=	Number of patients with biallelic variations		Number of patients with heterozygous variations			Patients without mutation
	*CYP24A1*	*SLC34A3*	*CYP24A1*	*SLC34A3*	*SLC34A1*	
	25	9	3	5	1	142
**serum calcium (mmol/L)**	**2.95 ± 0.11** (25)	**2.43 ± 0.10** (3)	2.94 ± 0.52 (9)	2.50 ± 0.04	2.97	2.88 ± 0.48 (132)
**serum phosphate (mmol/L)**	1.11 ± 0.08 (21)	1.14 ± 0.12 (3)	1.49 ± 0.18 (7)	1.30 ± 0.11 (5)	1.7	1.65 ± 0.04 (116)
**serum phosphate (SD)**	**-1.0 ± 0.4** (21)	**-2.3 ± 1.2** (3)	-1.0 ± 0.5 (7)	-1.0 ± 0.3 (5)	-1.3	-0.8 ± 0.1 (116)
**serum PTH (pg/mL)**	**6.8 ± 0.8** (25)	**8.0 ± 0.6** (3)	**8.4 ± 1.8** (8)	**16.1 ± 1.4** (5)	5	9.3 ± 0.5 (134)
**25-OH-D (nmol/L)**	**114.3 ± 11.5** (22)	**52.3 ± 8.8** (3)	91.6 ± 27.6 (9)	85.6 ± 28 (5)	130	88.7 ± 6 (122)
**1,25-(OH)_2_D (pmol/L)**	195.3 ± 22.6 (21)	234.1 ± 24.6 (3)	202.4 ± 43.2 (9)	243.2 ± 76.8 (3)	188	256.2 ± 15.2 (97)

In bold, statistically significant differences.mean ± standard error of mean (number of values).

Serum calcium in patients with biallelic *SLC34A3* mutation was lower than in patients with biallelic *CYP24A1* mutation (p=0.0123*). Serum 25-OH-D in patients with biallelic *CYP24A1* mutation was higher than in patients with biallelic *SLC34A3* mutation (p=0.0139*). There was no other statistically significant difference between these two groups of patients. Serum phosphate (SD) tended to be lower in patients with biallelic *SLC34A3* mutation than in patients with biallelic *CYP24A1* mutation.

#### Patients With Mutation in a Heterozygous State

Mutations in a heterozygous state were found in patients with renal stones (n=6), nephrocalcinosis (n=4) and no renal complications (n=5), mostly in *CYP24A1* (n=9) and *SLC34A3* (n=5).

Including class III variations, we identified 11 patients with a *SLC34A1* variation (7 of them carrying the recurrent c.272_292del/p.Val91_Ala97del) and 3 with a *SLC34A3* variation. Among this 14 patients with a heterozygous class III variation, 6 had nephrocalcinosis, 1 nephrolithiasis and 7 no renal disease. Especially, 3 patients with a heterozygous c.272_292del/p.Val91_Ala97del had nephrocalcinosis.

Serum PTH of probands with a *SLC34A3* mutation in a heterozygous state was higher than serum PTH in patients with biallelic *SLC34A3* and *CYP24A1* mutations (p=0.0357* and p=0.0003*** respectively). Biochemical profile of relatives and probands with a *SLC34A3* mutation in a heterozygous state did not differ statistically, although PTH tended to be higher and 25-OH-D and 1,25-(OH)_2_D tended to be lower in relatives. We observed typical HVD biochemical features in *SLC34A3* relatives’ group, namely hypercalciuria in 8/11 cases, low serum PTH in 6/11 cases, high 1,25-(OH)_2_D in 9/11 cases. Similarly, 2/4 relatives carrying a *SLC34A1* class III variation presented biochemical changes, 1 having a high urinary calcium excretion and 1 having hypophosphatemia (<-2 Z-score).

## Discussion

This report presents extensive clinical, biochemical and molecular data on a large cohort of patients with HVD phenotype and suspicion of IH1. We found: i) Mutations in genes associated with renal phosphate wasting can be found with a lower frequency than loss-of-function mutations in *CYP24A1* and digenism may represent a rare mechanism of HVD. ii) Our data indicate that hypophosphatemia is not specific to phosphate wasting defects, also observed in *CYP24A1* mutation, explaining a wide phenotypic overlap.

In our population, we found biallelic variants in *CYP24A1*, *SLC34A1* and *SLC34A3*, in accordance with an autosomal recessive inheritance, in 28-36 patients (15-19%), but no mutation in *SLC9A3R1*, suggesting that mutations in this latter gene do not cause HVD phenotype. We observed a majority of biallelic mutations in *CYP24A1*, in accordance with our inclusion criteria (low serum PTH and medical history of hypercalcemia and/or hypercalciuria). We found a high number of variants of uncertain significance in *SLC34A1* and *SLC34A3* (17/24) with difficulties interpreting their pathogenicity in a context of routine molecular diagnosis. Even the presence of the recurrent c.272_292del/p.Val91_Ala97del variant previously described in IH2 may still be difficult to interpret because of a high allelic frequency in general population and in our cohort, suggesting a likely benign variant rather than a pathogenic loss of function one. Although its biological activity was preserved, it had been reported as a pathogenic variant responsible for cellular localization defect (18, 37). Its association with another variant in *SLC34A1* in several patients (18) cannot exclude pathogenicity, maybe as an hypomorphic allele. Interpretation was more complex considering the high number of rare variants reported in a heterozygous state in these genes in online population databases (i.e. more than 700 *SLC34A3* variations in GnomAD). *In vitro* functional studies are not feasible in the context of routine diagnosis, justifying the need for specific clinical screening or diagnostic criteria and the collection of molecular data.

Most patients with biallelic variants presented with renal complications including chronic hypercalciuria, renal stones (6/28) or nephrocalcinosis (18/28); and were adults or children over 2 years old (24/28). The lower diagnosis rate in children (8-12% *versus* 45-51%) suggested that most of the children in our cohort may present with another disease or a transient HVD which may be due to the initial prematurity in renal function during early infancy (3). Indeed, relative renal immaturity may lead to a delay in the adaptation of mineral metabolism from a prenatal to a postnatal context, especially regarding the vitamin D 24-hydroxylase expression. Patients with biallelic variants in *CYP24A1* and *SLC34A1* presented a significant phenotypic overlap. However, these two disorders can be easily differentiated on the basis of the serum 25-OH-D_3_:24,25-(OH)_2_D_3_ ratio, which is distinctly elevated in patients with biallelic *CYP24A1* variants reflecting the vitamin D 24-hydroxylase defect; whereas the ratio remains normal in patients with biallelic *SLC34A1* variants (8–10). More comprehensive profiling of serum vitamin D metabolite profiles by LC-MS/MS including 23- and 24-hydroxylated metabolites (23,25,26-(OH)_3_D_3_, 25-OH-D_3_-26,23-lactone and 1,24,25-(OH)_3_D_3_), strengthen the differences in vitamin D metabolism in these two diseases, as well as other causes of vitamin D-related hypercalcemia (38). A few clinical criteria may also help distinguish *CYP24A1* mutation from other diseases. The presence of prenatal hyperechogenic kidneys in 2 patients of this cohort with *SLC34A1* class III variants and 2 patients in the literature (21) suggested a more precocious disease of the urinary tract. To the best of our knowledge, no patient with prenatal hyperechogenic kidneys and *CYP24A1* mutation were mentioned in the literature or observed in our patients. Mutations in *SLC34A3* are responsible for ARHR, with characteristic high urine calcium and serum 1,25-(OH)_2_D, but hypercalcemia in these patients was not documented to date. Among patients with biallelic *SLC34A3* variations, we reported 2 patients with fortuitously discovered hypercalcemia: A patient (n°152) with initial clinical features of HVD whose clinical presentation evolved into classic ARHR (with short stature and legs bowing); and a patient (n°88) with HVD with nephrocalcinosis without bone disease nor hypophosphatemia and normal height. These observations widen the phenotypic spectrum of *SLC34A3* mutations to HVD. Whether the HVD presentation corresponds to a sustainable mild form of phosphate tubular reabsorption defect or a precocious presentation of ARHR requires further investigation.

In our cohort, only 2 patients with biallelic variations in *SLC34A3* (class V) or *SLC34A1* (class III) presented with hypophosphatemia. Thus, most of them did not present with a biochemical profile suggestive of a phosphate wasting disorder *prima facie*, including 2 patients with biallelic *SLC34A3* pathogenic variations. Similarly, approximately one third of patients (5/16) did not present with hypophosphatemia in the IH2 cohort published by Schlingmann et al. (18) and patients with biallelic mutations in *SLC34A3* and normal serum phosphate have also been published (24). Case reports have thus suggested that supplementation with oral phosphate could help correct calcium metabolism in patients with *SLC34A1* mutation (18). More surprisingly, 27-28% of our patients with *CYP24A1* biallelic mutations also presented with hypophosphatemia.

It is important to consider these findings in relation to the role of FGF23 in HVD, also described by Brancatella et al. (18, 39). FGF23 is a key regulator of phosphate and vitamin D homeostasis, up-regulated by serum phosphate and 1,25-(OH)_2_D (40, 41). Binding of FGF23 to FGF receptors induces the retrieval of renal phosphate transporters (responsible for phosphate tubular reabsorption) and decreases the expression of vitamin D 1α-hydroxylase (*CYP27B1*) (42). In IH1, we suggest that reduced clearance of 1,25-(OH)_2_D up-regulates FGF23 production and consequently leads to phosphate wasting and hypophosphatemia. In contrast, in NaPiIIa/NPT2a/NaPiIIc/NPT2c defects, renal phosphate wasting is responsible for a decrease in serum phosphate which in turn down-regulates FGF23 and up-regulates 1α-hydroxylase. As a consequence, the increase in 1,25-(OH)_2_D favors phosphate and calcium intestinal absorption. The negative feedback exerted by the resulting increase in serum calcium is supported by the low serum PTH. Such mechanism had previously been proposed and corroborated by murine Slc34a1^-/-^ studies, illustrating the crucial role of FGF23 as a key regulator of vitamin D metabolism (18). Measurement of FGF23 in serum of HVD patients may be useful in future studies to specify this mechanism.

In 15-25 patients, we identified heterozygous variants, which represented diagnostic challenges in the context of a classic autosomal recessive disease. We could not exclude a second undetected variant (e.g. localized in non-coding sequences or a complex rearrangement) or contributory effect of the heterozygous variant as a risk factor. Although an autosomal dominant trait (7) had been proposed for heterozygous *CYP24A1* loss-of-function mutation (43), our previous work showed that heterozygotes (proband as well as relatives) had normal 24-hydroxylase activity (9, 13). Thus, a patient with a *CYP24A1* mutation in a heterozygous state and a high 25-OH-D_3_:24,25-(OH)_2_D_3_ ratio may benefit from extensive genetic exploration. Considering heterozygous variations in other genes, we observed a typical HVD phenotype in *SLC34A3* heterozygous relatives which had previously not been diagnosed as HVD patients. Similarly, it was suggested that *SLC34A1* heterozygous mutation may be a risk factor for kidney stone disease (18, 44) and heterozygous relatives may have abnormal biochemical data. It is likely that heterozygous phenotype may depend on additional factors, including genetics and environmental factors. Indeed, families in which the probands harbored a variation in two different genes suggested that heterozygous variations in *CYP24A1*, *SLC34A1* and *SLC34A3* may have an impact on vitamin D metabolism with a deleterious synergistic effect as previously proposed (45), as illustrated by the lower serum PTH in these patients compared to their heterozygous parents. Such a digenic mechanism, with both heterozygous *SLC34A1* and *SLC34A3* mutations, has been postulated as the cause of hypophosphatemic rickets with renal stone disease in an American family (46).

No mutation was identified in 66-77% of this cohort, especially in children (n= 110-126/150, 73-84%; adults: n=12-16/35, 34-46%). In our hands, these differences between children and adults may rely on obvious differences in the aims of genetic analysis in children and adults. In most children (78% or 117/150 children under 2 years), the first aim was to quickly distinguish *CYP24A1* mutations from other causes with transient HVD. We may wonder if these children had consistent phenotype over the years but this work does not include follow up data. Repeated evaluation of mineral metabolism including PTH after initial discontinuation of vitamin D supplementation, may be useful to detect a normalization of serum PTH (not in favor of a genetic defect) or a consistent low serum PTH (in favor of a genetic defect). Evaluation of 24-hydroxylated vitamin D metabolites may also represent an interesting tool in this purpose (8, 10, 38). On the other hand, genetic analyses in adult were conducted in a different context of longer medical history to explain a phenotype. We cannot assume that patients without mutation in *CYP24A1*, *SLC34A1*, *SLC34A3* or *SLC9A3R1* did not carry variations in other genes or regulating sequences which were not analyzed.

No mutation was identified in *SLC3A3R1* encoding NHERF1 in our cohort, which may be explained by our inclusion criterion as serum PTH in patients with *SLC3A3R1* mutation were above 20 pg/mL in previous publications (25, 26).

Unfortunately, unlike genetic data obtained from the same procedure, the data collected in this retrospective cohort may include bias due to the aggregation of biochemical results from various assays and routine medical laboratories, which may impact the interpretation of 1,25-(OH)_2_D particularly. Appropriate exploration of phosphate metabolism including serum FGF23 evaluation would be of great interest to further characterize this pathophysiologic mechanism in HVD. Lastly, several patients without mutation (n=24) presented with hypophosphatemia suggesting the existence of other different mechanisms of calcium, vitamin D and phosphate metabolism deregulation.

## Conclusion

With the exception of *CYP24A1*, specific biochemical profiles associated with individual candidate gene are lacking, making diagnosis of HVD patients challenging. Our results emphasize the importance of assessing *SLC34A1* and *SLC34A3* during genetic exploration of HVD in patients without vitamin 24-hydroxylase deficiency. While personalized treatment approaches for HVD such as hydrochlorothiazide, ketoconazole and fluconazole, phosphate oral supplementation have been described; our findings are anticipated to assist physicians with rapidly arriving at the correct diagnosis in the context of overlapping phenotypes in HVD patients so that appropriate treatment can be given.

## Data Availability Statement

The datasets presented in this study can be found in online repositories. The names of the repository/repositories and accession number(s) can be found in the article/**Supplementary Material**.

## Ethics Statement

The studies involving human participants were reviewed and approved by CPP Nord Ouest III. Written informed consent to participate in this study was provided by the participants’ legal guardian/next of kin. Written informed consent was obtained from the individual(s), and minor(s)’ legal guardian/next of kin, for the publication of any potentially identifiable images or data included in this article.

## Author Contributions

AM and M-LK designed the study. CB and NC realized molecular study. AM, NR, and HM interpreted molecular study. AM, SL, JB, PB, AL, and MN collected the clinical and biochemical data. MK and GJ realized LC-MS/MS studies. AM analyzed the data, drafted the paper and made all tables, and figures. M-LK, JB, SL, GJ, and MK revised the paper. All authors contributed to the article and approved the submitted version.

## Funding

Délégation Recherche Clinique et Innovation (DRCI) - CHU de Caen APRIM - 16-048.

## Conflict of Interest

The authors declare that the research was conducted in the absence of any commercial or financial relationships that could be construed as a potential conflict of interest.

## Publisher’s Note

All claims expressed in this article are solely those of the authors and do not necessarily represent those of their affiliated organizations, or those of the publisher, the editors and the reviewers. Any product that may be evaluated in this article, or claim that may be made by its manufacturer, is not guaranteed or endorsed by the publisher.

## Supplementary Material

Supplementary Table 1 Raw dataset.
